# Nicotine suppresses Parkinson’s disease like phenotypes induced by Synphilin-1 overexpression in *Drosophila melanogaster* by increasing tyrosine hydroxylase and dopamine levels

**DOI:** 10.1038/s41598-021-88910-4

**Published:** 2021-05-05

**Authors:** Angel Carvajal-Oliveros, Carmen Domínguez-Baleón, Rafaella V. Zárate, Jorge M. Campusano, Verónica Narváez-Padilla, Enrique Reynaud

**Affiliations:** 1 Departamento de Genética del Desarrollo y Fisiología Molecular, Instituto de Biotecnología, Universidad Nacional Autónoma de México, UNAM, A.P. 510-3, 62210 Cuernavaca, Mor. Mexico; 2 ﻿Laboratorio Neurogenética de la Conducta, Departamento de Biología Celular y Molecular, Facultad de Ciencias Biológicas, Pontificia Universidad Católica de Chile, Santiago, Chile; 3 Centro de Investigación en Dinámica Celular, Universidad Autónoma del Estado de Morelos, Cuernavaca, Morelos Mexico

**Keywords:** Biochemistry, Neurochemistry, Neuroscience, Cell death in the nervous system, Diseases of the nervous system, Genetics of the nervous system

## Abstract

It has been observed that there is a lower Parkinson’s disease (PD) incidence in tobacco users. Nicotine is a cholinergic agonist and is the principal psychoactive compound in tobacco linked to cigarette addiction. Different studies have shown that nicotine has beneficial effects on sporadic and genetic models of PD. In this work we evaluate nicotine’s protective effect in a *Drosophila melanogaster* model for PD where Synphilin-1 (Sph-1) is expressed in dopaminergic neurons. Nicotine has a moderate effect on dopaminergic neuron survival that becomes more evident as flies age. Nicotine is beneficial on fly survival and motility increasing tyrosine hydroxylase and dopamine levels, suggesting that cholinergic agonists may promote survival and metabolic function of the dopaminergic neurons that express Sph-1. The Sph-1 expressing fly is a good model for the study of early-onset phenotypes such as olfaction loss one of the main non-motor symptom related to PD. Our data suggest that nicotine is an interesting therapeutic molecule whose properties should be explored in future research on the phenotypic modulators of the disease and for the development of new treatments.

## Introduction

PD is the second most common neurodegenerative disorder and it is characterized by the loss of dopaminergic neurons of the *sustantia nigra pars compacta*. Neuron cell death diminishes the amount of dopamine released in the *striatum* and other parts of the brain leading to the characteristic motor symptoms associated with this disease. Non-motor symptoms are also observed, including loss of olfaction and sleep disorders^1–3^. In classic PD, is possible to find intracellular protein inclusions in the soma of the surviving dopaminergic neurons, known as Lewy bodies^4^. The primary component of these aggregates is α-Synuclein (α-Syn), a protein vastly found in pre-synaptic terminals, which has been proposed to be involved in assembly of the SNARE complex and neurotransmitter release^5–7^. α-Syn monomers interact with each other to form oligomers and promote the accumulation of other proteins. Proteomic assays have shown a physical interaction of α-Syn with at least another 571 proteins, among which the most important are the PD associated proteins PINK1, LRKK2 and Sph-1^8^.

Sph-1 has been involved in the progression of PD and it is also one of the most abundant components of Lewy bodies**.** Human Sph-1 is a 919 amino acid cytoplasmic protein codified by the gene SNCAIP. Sph-1 inhibits the E3 ubiquitin ligase activity of SIAH1 and the proteasomal degradation of several proteins including its own^9^. Sph-1 appears to be important for synaptic connections but its specific functions, just as those of α-Syn, are unknown^10^. It has been proposed, that Sph-1 might be involved in supporting proteasome degradation, and given its strong interaction with α-Syn, it could play a central role in Lewy body formation and synaptic function^11,12^. Sph-1 has at least eight isoforms and some of them lack the N-terminal domain, a feature known to promote its aggregation^13–15^.

Currently, there are only symptomatic treatments for PD. Therefore, it is of crucial importance to search for molecules that might help maintain dopaminergic neuron function or prevent degeneration caused by protein accumulation^16^. Remarkably, one of these potentially protective molecules is nicotine, the primary psychoactive substance of tobacco. Epidemiologic research has shown that tobacco use may reduce up to 50% the risk of suffering PD^17–19^. Although nicotine is known as an acetylcholine receptor agonist that promotes cellular excitability, the mechanisms underlying its cytoprotective mechanisms are unknown^20^.

Here, we evaluated the effect of a chronic nicotine treatment on a *Drosophila melanogaster* model for PD generated by the expression of human Sph-1 in dopaminergic neurons. Our results show that expression of Sph-1 in *Drosophila* central nervous system (CNS) neurons promotes characteristic Parkinsonian phenotypes such as motor impairment, neuronal death and dysregulation of the dopaminergic neural system. Remarkably, the fly PD model also exhibits olfactory dysfunction in young flies, probably the most important early-onset, non-motor symptom of the disease. Nicotine treatment has a beneficial effect on survival, motility and dopamine levels in flies that express Sph-1 and are therefore predisposed to dopaminergic neurodegeneration. On the other hand, nicotine has deleterious effects in control flies. These data suggest that Sph-1 expression in *Drosophila* CNS neurons is a good model to study the progression of PD through aging. The demonstration that nicotine counteracts several PD phenotypes, makes this molecule a very interesting seed compound for the search of new chemicals against PD.

## Methods

### Genotypes and crosses

All behavioral experiments were performed with male flies. Flies were fed with standard cornmeal-yeast food and kept at 25 °C in an incubator under 12/12 light/dark cycle. *UAS-GFP, th-GAL4* and *w*^*1118*^ were obtained from the Bloomington Drosophila Stock Center. *UAS-Sph* was generated in our laboratory and expresses the canonical 919 amino acid long isoform 1 as reported in the UniProt database (https://www.uniprot.org/uniprot/Q9Y6H5)^21^. Experimental genotype was *UAS-GFP/UAS-Sph; th-GAL4/*+. Control flies (*UAS-GFP/*+*; th-GAL4/*+*)* were in all cases the progeny of the *UAS-GFP/UAS-GFP; th-GAL4/th-GAL4* crossed with *w*^*1118*^.

### Nicotine treatment

Standard cornmeal-yeast food was supplemented with 24 µM nicotine base. Flies were transferred to fresh food vials supplemented with nicotine every third day for the duration of the experiment.

### Life expectancy and motility assays

Groups of 20 one-day old male flies were placed in standard corn meal agar food with or without nicotine (experimental and control groups, respectively) at the corresponding concentrations. Animals were transferred to fresh vials every 4 days; dead, trapped and escaped individuals were recorded (n = 100 per group). Assays were performed at 25 °C under 12/12 h light/dark cycle. Parameters analyzed include the maximum life span (the time point at which no alive flies are found), and the half lifespan (the time point where 50% of flies have died). In the discontinuation of chronic nicotine treatment or late in life treatment initiation experiments, an initial cohort of 200 individuals of the corresponding genotypes was separated in two groups of 100 individuals each: one group was treated with nicotine only for the first half of its life-expectancy (first 60 days after eclosion), while the other was treated only for the second half of its life expectancy (treatment initiated only after 60 days of aging); both groups were reared an analyzed simultaneously for their whole life-span until there were no survivors.

Negative geotaxis was assayed essentially as reported by Ali^22^; flies were aged and their climbing was measured every 5 days until they were 60 days old. Five vials with 10 flies of the corresponding genotype were analyzed. Flies were forced to the bottom of the vial with three gentle taps. Then, their ability to climb over 10 s to a 5 cm mark was quantified. Animals were allowed to rest for a minute and then the assay was repeated with the same population. This procedure was repeated ten times in total.

Spontaneous activity was quantified using the *Drosophila* Activity Monitor (*TriKinetics*)^23^; 14 individuals of each genotype, age and treatment were allowed to adapt for 24 h to the setup, and afterwards data was recorded for 3 days in a 12 h light/dark cycle. Activity was analyzed separately for the light and dark periods.

### Olfactory assays

Olfactory test was analyzed using the BuriTrack software^24^. Briefly, individual flies of each genotype and treatment were placed on a circular white arena (39 mm wide 2 mm tall). Aversive olfactory stimulus (100 µl 1% benzaldehyde -Bz- in water on a small cotton ball) and control (100 µl distilled water on a small cotton ball) were presented through 0.1 mm holes on opposite sides of the arena. The preference of flies for either side of the arena was quantified according to Molina-Mateo et al.^25^. The performance index is reported (n = 15 for each genotype and treatment).

### Neuronal survival quantification

GFP expression in *UAS-GFP/*+*; th-GAL4/*+ organisms were used as a reporter for surviving dopaminergic neurons. Neuronal survival was quantified by dissecting out brains of the corresponding age and treatment. Brains were fixed with 4% formaldehyde in PBS, mounted individually in Citifluor (Ted Pella Inc. Redding, CA.) and images were taken at the National Laboratory of Advanced Microscopy using a confocal Olympus FV10 microscope with a 20X objective. Five brains of each genotype, age and condition were counted in a blinded manner. Confocal acquisition parameters were set and fixed using 10 days old control flies (UAS-GFP*/*+:Th-GAL4/+). the whole brain was sampled avoiding pixel saturation in the brightest section with optimal pinhole aperture and optimal section thickness, steps were 0.5X section thickness so the whole tissue was sampled twice, after data collection, maximum intensity projections (assembled with image J, https://imagej.nih.gov/ij/ taking in account section thickness and 2X oversampling) were used to make a single flat image and neurons were counted, if there was ambiguity about the number of neurons in a particular cluster in a particular maximally projected brain image, individual sections were analyzed. All other conditions and genotypes were acquired using these pre-set acquisition parameters.

### Dopamine quantification

Total brain dopamine was quantified as in Molina-Mateo et al.^25^ using HPLC (BAS, West Lafayette, IN) coupled to electrochemical detection. Briefly, adult brains of the corresponding age and treatment were dissected, homogenized using sonication in 0.2 N Perchloric acid and the solution filtrated through a 0.2 µm filter. 5 µl of the extracts were injected in the HPLC system with the following configuration: a pump (model PM-80), a SepStick microbore column, and an amperometric detector (model LC-4C). The HPLC mobile phase, consisting of 0.1 M NaH_2_PO_4_, 1.8 mM 1-octanesulfonic acid and 1 mM EDTA (pH adjusted to 2.3) was pumped at a flow rate of 60 μl/min. The potential of the amperometric detector was set at 0.650 V. Under these experimental conditions, retention times were 5.50 and 6.80 min for L-DOPA and dopamine, respectively. Samples were analyzed by comparing the peak area of the neuroactive molecules and their elution times with respect to reference standards. The detection limit for L-DOPA and dopamine was 0.5 fmol/μl, allowing measurement of baseline levels.

### Western blot densitometry

Total head extracts were used for semiquantitative densitometric Western blot. Briefly, 50 µg of protein were loaded in 12% Laemmli-SDS-PAGE gels (Biorad Mini protean gel electrophoresis system) and transferred to nitrocellulose membranes for 3 h at 250 mA. Membranes were blocked overnight at 4ºC temperature with PBS 0.1% tween (PBST) supplemented with 10% milk. Membranes were then incubated at 4 °C overnight with antiTH (Inmunostar 1:1000) in 5% milk PBST, washed 3 times with PBST and incubated at room temperature for 1 h with KPL HPRT goat-antimouse (Sera Care) in 5% milk PBST. Blots were then washed 3 times in PBST, and bands were detected using the SuperSignal West pico Chemiluminiscence Substrate (ThermoFisher) according to manufacturer’s instructions. Blot signals were digitalized and quantified using ImageJ. ﻿Mouse monoclonal anti-actin (DHB, 1:3000) was used as loading control.

### Statistical analysis

Differences between groups were assessed using ANOVA and Tuckey’s pot-hoc test. Survival (life expectancy) was analyzed using the Mantel-Cox log-rank test. Survival of aged flies (older than 80 days of age) was analyzed using Fisher’s exact test. Average data is plotted with its corresponding Standard Error, for each experiment the number of individual experiments is shown. Significance was defined as *p* < 0.05. All data was analyzed using GraphPad Prism 8 software.

### Ethical approval.

No human or vertebrates were used in this works. The project was approved by the Bioethical Committee of the Instituto de Biotecnología and performed accordingly to the ethical guidelines of our institution.

## Results

### Determination of optimal nicotine dosage

We determined an optimal nicotine concentration that was the least harmful to wt flies and the one that had the most beneficial therapeutic effect in the experimental flies through a set of preliminary dose–response experiments (Supplementary Fig. 1) that consisted in long term survival experiments in food supplemented with fixed doses of nicotine base (12, 24, and 48 µM). These doses were equivalent and had a similar effect to the ones used by Chambers et al*.*^26^. All subsequent experiments were performed at 24 µM after determining that this was the optimal concentration. Even though, this nicotine concentration may superficially appear to be high, it is in the lower range of the concentrations used in other animal models such as mice and zebra fish that tend to be a lot higher. In mice, nicotine experimental concentrations range from 25 to 400 µM^27^. In zebra fish where the actual absorption is much higher because nicotine is supplemented in the water where the fish swim and it is absorbed through the skin, the range of nicotine concentrations used is from 5 to 100 µM^28^. In any case, the amount of nicotine fed to the flies is not the actual concentration reaching tissues and organs. In our experience, when animals are fed a chemical, the actual concentration that reaches the internal organs is about 1/100 of the original dose^29^. Thus, given that the concentration of nicotine chosen in our study for feeding experiments is 24 µM, it is reasonable to estimate that the actual concentration in the hemolymph or the fly brain is about 200 nM. Interestingly, it has been established that in humans, the peak concentration of nicotine in the blood after smoking one cigarette is about 300 nM^30^ which is a very similar concentration to the one estimated to reach the brain in our experiments.

**Figure 1:**
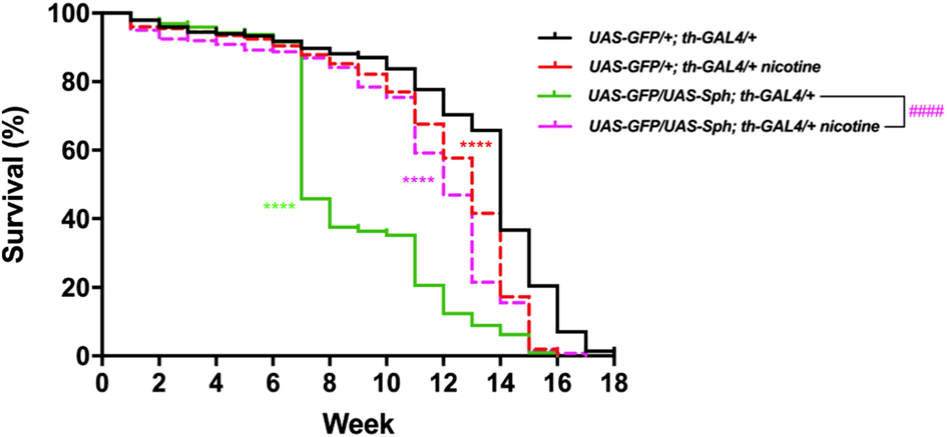
Nicotine treatment increases lifespan in Sph-1 expressing flies, while reduces it in control animals. Lifespan in control animals (that do not express Sph-1) is reduced when treated with 24 µM nicotine. Flies expressing Sph-1 in dopaminergic neurons have a significantly reduced life span (green solid line). However, this is rescued by chronic nicotine treatment (magenta dotted lines). Data of untreated control animals are presented with black solid lines; data of nicotine-treated control animals are graphed with red dotted lines. Statistical analyses were performed using Log-rank (Mantel-Cox) test. Asterisks are used to show statistical significance between the untreated control line (*UAS-GFP/*+*; th-GAL4/*+) and all the other genotypes and conditions, the number symbol (#) is used to show statistical significance between the treated and untreated Sph-1 lines (*UAS-GFP/UAS-Sph; th-GAL4/*+) **** or ####*P* < 0.0001, n = 200 animals per experimental condition. All treated animals were chronically exposed to 24 µM nicotine.

### Nicotine promotes survival in flies that express Sph-1

**Figure 2:**
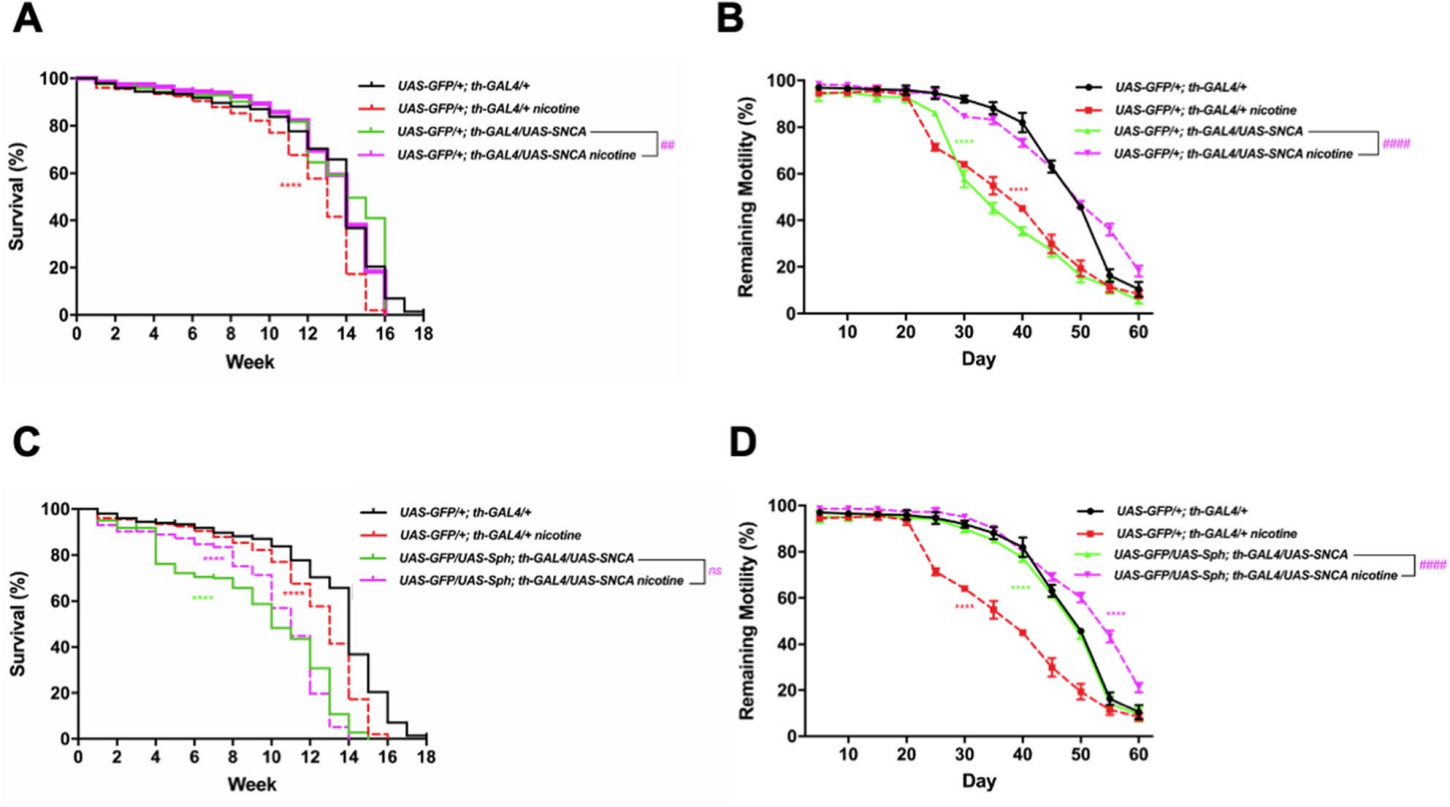
Nicotine treatment increases motility in α-synuclein (SNCA) expressing flies, while reduces it in control animals. (**A**) Lifespan and motility in control animals (that do not express SNCA) are reduced when treated with 24 µM nicotine. Flies expressing SNCA in dopaminergic neurons have a significantly reduced life expectancy which is similar in chronic nicotine treated animals (24 µM). (**B**) However, motility is rescued in genotypes that are predisposed to PD like symptoms and treated with nicotine. (**C**,**D**) show the lifespan and motility in animals that co-express Sph-1 and α-Syn. As previously reported ^21^, animals that co-express both proteins have a milder phenotype that the ones that only express Sph-1. It has previously been reported that the co-expression of Sph-1 with α-Syn partially suppresses the toxicity caused by the expression of the individual proteins. The flies that co-express Sph-1 and α-Syn have milder phenotypes, and the effect of nicotine is also more subtle. Regardless of the penetrance of the PD like genotype, nicotine always appears to have beneficial therapeutic effects in the organisms that are predisposed to PD like symptoms. Data of untreated control animals are presented with black traces solid lines; data of nicotine-treated control animals are graphed with red traces dotted lines. Statistical analyses were performed using Log-rank (Mantel-Cox) test. Asterisks are used to show statistical significance between the untreated control line (*UAS-GFP/*+*; th-GAL4/*+) and all the other genotypes and conditions, the number symbol (#) is used to show stratistical significance between the treated and untreated SNCA lines (*UAS-GFP/*+ *; th-GAL4/UAS-SNCA*) **** or ####*P* < 0.0001, ###*P* < 0.001, ns: no aignificative. n = 200 animals per experimental condition. All treated animals were chronically exposed to 24 µM nicotine.

We defined the most effective chronic dose of nicotine on the lifespan of flies that have been previously used as a model for PD (*UAS-GFP/UAS-Sph; th-GAL4/*+)^21^ (Fig. 1 and Supplementary Fig. 1). Flies that co-express Sph-1 and GFP in their dopaminergic neurons exhibited a half-life of 7 weeks. Nicotine treated flies of the same genotype had a significant increase in their maximum life span from 16 to 17 weeks and also in their half-life from 7 to 13 weeks (Fig. 1). As a control genotype we used *UAS-GFP/*+*; th-GAL4/*+ that expresses GFP, a protein unrelated to PD. Interestingly, the maximum lifespan and half life expectancy of nicotine treated control flies were significantly reduced from 18 to 16 weeks, and from 14 to 13 weeks respectively (Fig. 1). We also tested the effect of nicotine treatment in flies that express α-synuclein and GFP in their dopaminergic neurons (*UAS-GFP/*+*; th-GAL4/UAS-SNCA*), these flies have milder and more subtle phenotypes. Consistent with our results with Sph-1 expressing flies, nicotine treatment significantly improved motility and survival parameters of the α-synuclein expressing flies while having a deleterious effect on the control genotype. It has previously been reported that the co-expression of Sph-1 and α-Syn partially suppresses the toxicity caused by the expression of either of the proteins alone. Interestingly, when we treated these flies (*UAS-GFP/UAS-Sph-1; th-GAL4/UAS-SNCA*) that have a milder PD-like phenotypes with nicotine, its effect still significantly improves their motility while slightly reducing maximal lifespan Fig. 2)^21^. These data support the idea that nicotine promotes survival only in flies predisposed to develop a PD-like phenotype.

### Nicotine rescues motility in flies that express Sph-1

**Figure 3:**
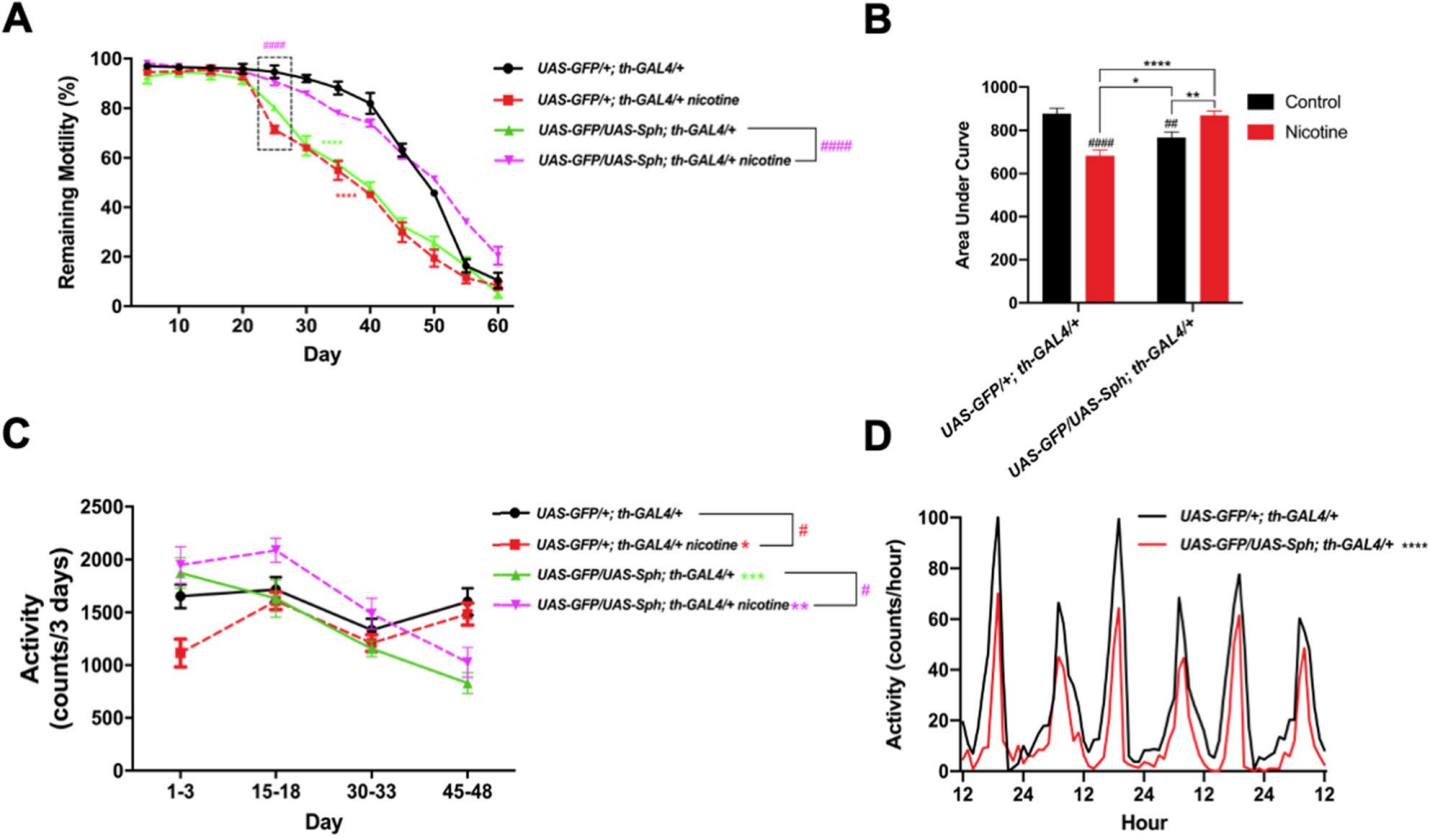
Nicotine rescues spontaneous and startle-induced motility in animals that express Sph-1 but deteriorates locomotion in control flies. (**A**) Startle-induced response of flies of the control genotype (*UAS-GFP/*+*; th-GAL4/*+) (black solid lines) exhibit a startle-induced response that is reduced when animals are treated with nicotine (red dotted lines). Startle-induced motility is significantly better when experimental genotype Sph-1 animals (*UAS-GFP/UAS-Sph; th-GAL4/*+) (green solid lines) are treated with nicotine (magenta dotted lines). (**B**) Cumulative locomotion in startle-induced experiments was assessed as area under the curve from data presented in (**A**). Results show that motor response is reduced in the Sph-1 expressing flies compared with the control genotype and that motility is rescued when experimental animals are treated with nicotine (## indicates *p* < 0.01 compared with control group). In addition, a significant decrease in motor response is observed in control flies treated with nicotine compared with flies that did not receive nicotine (#### indicates *p* < 0.0001 compared with control situation). (**C**) Control animals (black solid lines) exhibit spontaneous activity that significantly decreases when flies are treated with nicotine (red dotted lines) while spontaneous activity is rescued in the Sph-1 expressing ones (magenta dotted lines). In (**A**–**C**), two-way ANOVA followed by Tukey’s post-hoc was used to evaluate statistical differences between experimental groups. In all conditions n = 100, * indicates *p* < 0.05; ** indicates *p* < 0.01; ****indicates *p* < 0.0001. Data from untreated flies are represented in dotted lines, while data from nicotine-treated animals are shown in solid lines. (**D**) Actograms of control and Sph-1 expressing flies. Data from control genotype are presented in black, data from Sph-1 expressing flies is shown in red. Two-way ANOVA followed by Tuckey’s post-hoc, ****indicates *p* < 0.0001 between groups, n = 14 animals per group. All treated animals were chronically exposed to 24 µM nicotine.

One of the most characteristic phenotypes of PD is the impairment in spontaneous and induced motility^31^. The startle-induced climbing assay (negative geotaxis) is a paradigm that has been used to study how locomotion is reduced as flies age. Consistent with this, our data show that the climbing ability of flies diminishes as they age (Fig. 3A,B). Interestingly, untreated flies expressing Sph-1 in their dopaminergic neurons showed significantly faster decay in the startle-induced climbing ability in comparison with control animals (Fig. 3A,B). In order to quantify this effect, we recorded the accumulated motility over time (expressed as Area Under Curve). Our data show that Sph-1 expressing flies (green solid lines) exhibit reduced motility compared with control animals (Fig. 3A black solid lines). Remarkably, nicotine treatment completely suppresses this phenotype in Sph-1 expressing flies (Fig. 3A magenta dotted lines, and 3B). On the other hand, control flies treated with nicotine showed a significant reduction in their climbing ability (Fig. 3A, red dotted lines and B).

Experiments aimed at evaluating spontaneous activity show similar results, being the effect only observable during the light periods. Animals expressing Sph-1 show reduced spontaneous motility in comparison with the control genotype, particularly at older ages (Fig. 3C,D). This can be better evidenced when looking at actograms of these two strains (Fig. 3D). On the other hand, the motor phenotype observed in Sph-1 expressing animals is partially suppressed by chronic nicotine treatment (Fig. 3C, magenta dotted lines). Control animals that do not express Sph-1 exhibit reduced spontaneous motility when treated with nicotine (Fig. 3C, red dotted lines). Spontaneous activity at dark periods is not different (Supplementary Fig. 2).

### Sph-1 expression induces impairment in olfaction in young flies

**Figure 4:**
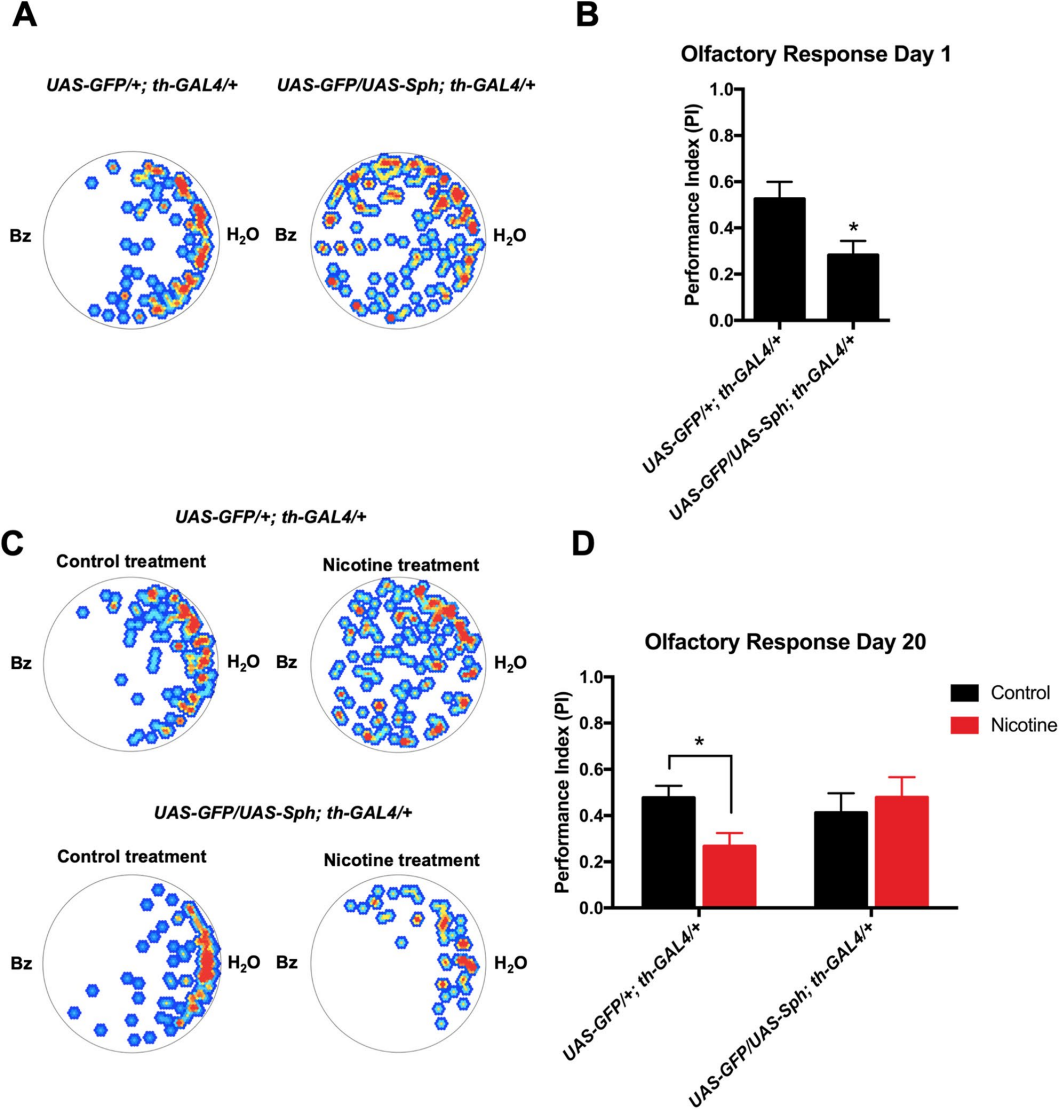
Young flies that express Sph-1 in dopaminergic neurons exhibit impaired olfactory responses. (**A**) Heat maps of a typical experiment showing the aversive response towards benzaldehyde (Bz) of recently eclosed flies of the control genotype (*UAS-GFP/*+*; th-GAL4/*+) and of flies expressing Sph-1 in dopaminergic neurons (*UAS-GFP/UAS-Sph; th-GAL4/*+). The position of flies over 3 min is presented in color ranges, where red or yellow hue represent points in the arena where flies spent longer time, while blue or white hue show regions in the arena where flies spent little or no time at all. In both cases, Bz was placed to the left side of the arena. **B)** Quantification of the cumulative behavior of the flies tested for each genotype expressed as Performance Index. These data shows that aversive response is reduced in Sph-1 expressing flies compared with control flies. **C)** Representative heat maps of behavior of flies (20 days old) from both genotypes, after chronic treatment with nicotine, or in control condition (no treatment). **D)** Quantification of behavior of several flies (20 days old) show no difference in olfactory response between the two genotypes in control condition. Aged control flies treated with nicotine show reduced olfactory response compared with control flies not receiving the cholinergic agonist. Data from untreated animals were represented in black bars; data from nicotine-treated animals were represented in red bars. *P < 0.05, n = 15 animals per condition. Two-way ANOVA. Tuckey’s post-hoc test. All treated animals were chronically exposed to 24 µM nicotine.

A recognized PD feature is early onset olfactory loss^32^. We decided to assess olfactory responses in the Sph-1 expressing flies. Newly eclosed (one day old) flies exhibit strong aversion towards the odorant benzaldehyde (1%), evidenced by a Performance Index close to 0.5. Figure 4A,B show that newly eclosed flies that express Sph-1 in their dopaminergic neurons have a significant reduction in their response towards Bz compared with control animals. If the animals are aged for 20 days no olfactory phenotype is observed in Sph-1 expressing animals compared with control flies. Chronic nicotine treatment reduced the aversive response to benzaldehyde in aged control animals while induced a slight but not significant increase in olfactory performance in aged Sph-1 expressing flies (Fig. 4C,D). These data supports the hypothesis that nicotine treatment negatively affects olfactory performance of control animals.

### Nicotine treatment increases tyrosine hydroxylase levels and brain dopamine content

**Figure 5:**
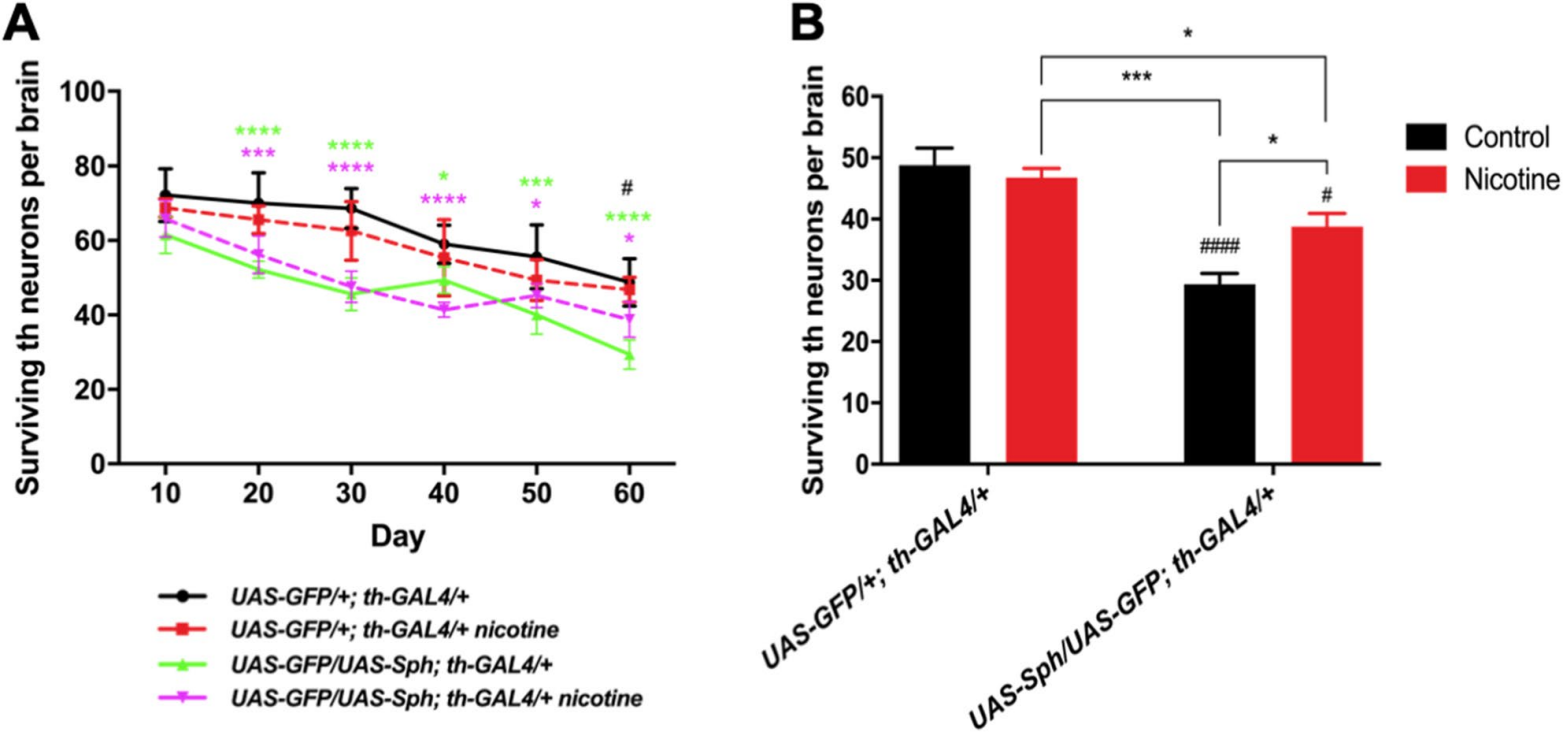
Nicotine promotes dopaminergic neuron survival. A) Dopaminergic neuron loss over time in control (UAS-GFP*/*+; th-GAL4/+) and experimental (*UAS-GFP/UAS-Sph; th-GAL4/*+) genotypes; untreated populations are represented by solid lines and treated populations by dotted lines. No significant differences were evidenced between the treated and untreated control genotype. Flies expressing Sph-1 in dopaminergic neurons have significantly fewer neurons than the control genotype throughout their life. However this difference becomes smaller between control and the Sph- expressing treated population as flies age. **B)** The number of surviving TH neurons in each experimental condition at the last time point studied (Day 60) evidences a reduction in th-positive neurons in the Sph-1 expressing flies compared with control genotype (#### p < 0.0001). Interestingly, this analysis also shows that nicotine treatment does not affect the number of th-positive neurons in the control genotype (p > 0.05), but it does so in the Sph-1 expressing flies (# p < 0.05 compared with the control genotype not treated with nicotine). The Sph-1 expressing flies treated with nicotine show a partial recovery in the number of th-positive neurons, since it is significantly increased compared with not-treated Sph-1 expressing flies (*p < 0.05), but does not reach the number of positive neurons recorded in the control genotype (*p < 0.05). In each case, data from controls were represented in black bars; data from animals treated with nicotine were represented in red bars. All statistical analysis were from two-way ANOVA followed by Tuckey’s post-hoc test, n = 5 fly brains. In all cases experimental animals were treated with 24 µM nicotine.

The nicotine effect on fly survival (Fig. 1) and motility (Fig. 3), might be explained by a modification in some of the functional properties of dopaminergic neurons, leading to cell survival and/or preserving neuronal function. We decided to explore both possibilities. We assessed the total number of dopaminergic neurons in the genotypes of interest and their change as flies age. We then evaluated whether nicotine treatment affects the number of surviving dopaminergic neurons in these animals. Our data show that the number of tyrosine hydroxylase positive neurons decrease in both genotypes, over aging (Fig. 5A,B and Supplementary Fig. 3). The number of positive cells decreases faster in animals expressing Sph-1 in their dopaminergic neurons than in control flies, which is evidenced by the slope of the curves. Importantly, the rate of dopaminergic neuron decay is slowed down by nicotine treatment in the PD fly model, an effect that is more evident at later time points (40 days old and older). The evaluation of the number of dopaminergic neurons in 60 days old flies, show that nicotine treatment significantly increases the number of surviving dopaminergic neurons in Sph-1 expressing animals (Fig. 5B). On the other hand, nicotine treatment did not have any effect on the rate of dopaminergic cell loss in control animals (Fig. 5A), which is also evidenced in their number of surviving dopaminergic cells (Fig. 5B). Thus, our data suggest that at least some of the effects induced by nicotine on survival and motor behavior, involve differential changes of this cholinergic agonist on the two genotypes.

**Figure 6:**
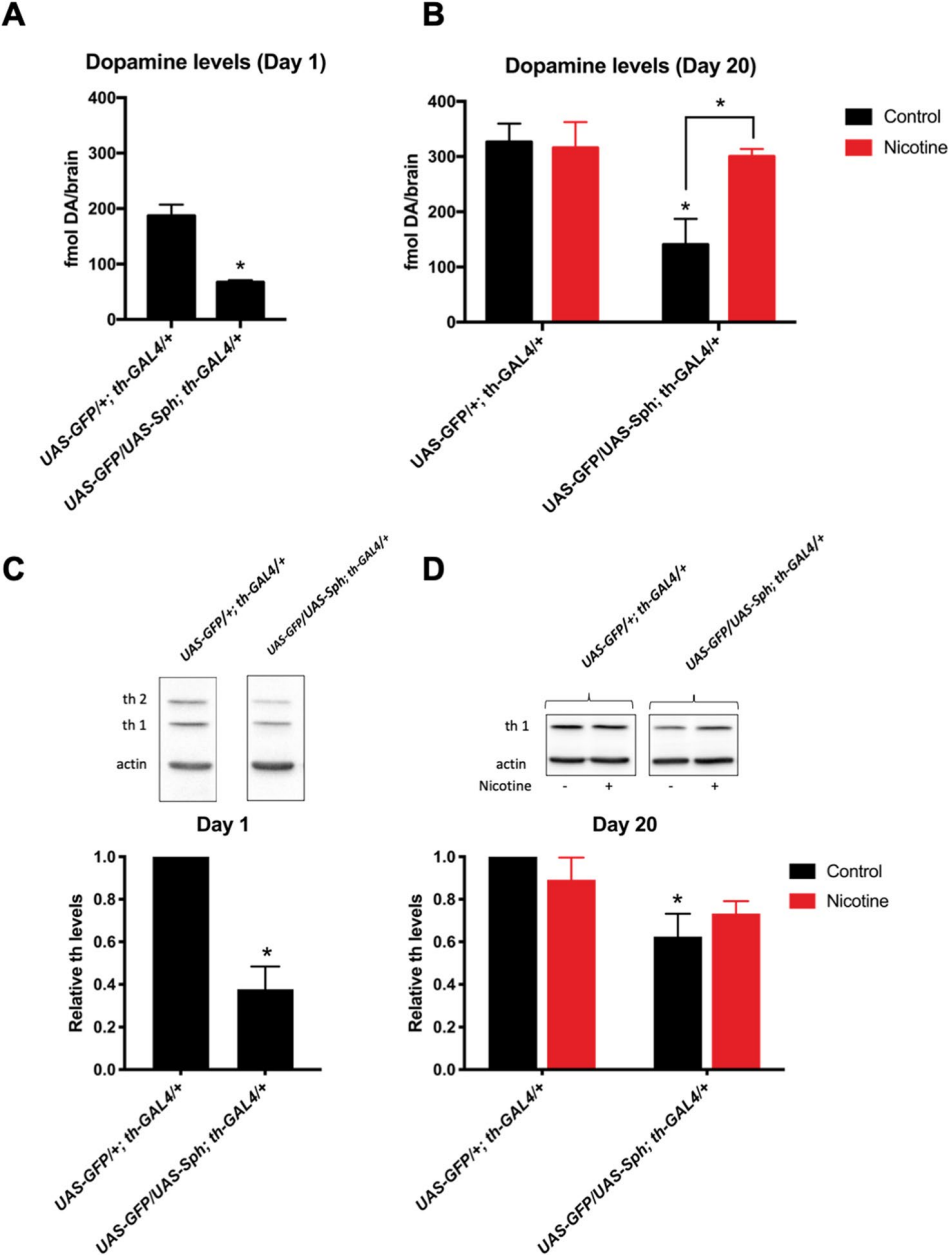
Nicotine exposure increases brain dopamine and tyrosine hydroxylase levels. (**A**) Newly eclosed flies (one day old) that express Sph-1 (*UAS-GFP/UAS-Sph; th-GAL4/*+) show reduced dopamine brain levels compared with control (UAS-GFP/+; th-GAL4/+) flies. *p < 0.05, t-test (**B**) After 20 days of aging, dopamine levels are reduced in animals that express Sph-1 compared with control flies (#*p* < 0.05). 20 days of chronic nicotine treatment induces an increase in dopamine brain levels in Sph-1 expressing flies (*p < 0.05) so amine brain content becomes similar to levels recorded in control flies. Dopamine levels in control animals are not affected by the nicotine treatment. In each case, data from controls were represented in black bars; data from animals treated with nicotine were represented in red bars. Statistical analysis after two-way ANOVA and Tuckey’s post-hoc test (n = 5 samples, each consisting of 5 fly brains). (**C**) At day one, tyrosine hydroxylase isoform 1 (th1) protein levels are reduced in flies that express Sph-1 (*UAS-GFP/UAS-Sph; th-GAL4/*+) when compared with control flies (UAS-GFP/+; th-GAL4/+), as can be observed in a representative experiment (top panel) in young flies. The higher MW cuticle-specific tyrosine hydroxylase isoform 2 (th2) can also be detected; this isoform stops being expressed as soon as the fly´s cuticle is melanized and cannot be detected in aged flies. Quantification of several blots from independent experiments (bottom panel) show significant reduction in tyrosine hydroxylase protein levels (th1). *P < 0.05; t-test compared with control strain; n = 3 different samples obtained from independent groups of animals. (**D**) At day twenty, untreated Sph-1 expressing flies show lower tyrosine hydroxylase levels compared with control animals, as evidenced in a typical experiment (top panel). Quantification of several blots from three independent biological samples (bottom panel) show a significant lower protein expression in Sph-1 expressing flies (*P < 0.05). Nicotine treatment promotes an increase of tyrosine hydroxylase to normal levels compared with the control genotype (*p* > 0.05). Statistical analyses with two-way ANOVA and Tuckey’s post-hoc test, in n = 3 independent samples. In all cases experimental animals were treated with 24 µM nicotine, full blots used for quantifications are shown in Supplementary Fig. 4.

We then decided to determine if nicotine can affect dopaminergic function later in life. In order to explore this possibility, we evaluated total brain dopamine content in young and aged adult *Drosophila* brains. Our results show that there is a decrease in total dopamine levels in newly eclosed flies that express Sph-1 in their dopaminergic neurons compared with control animals (Fig. 6A). The difference in brain dopamine content observed between the experimental and control strains can still be observed in twenty days old flies (Fig. 6B). Chronic nicotine treatment significantly increases the dopamine levels in Sph-1 expressing flies to control levels. However, brain dopamine levels in control animals are not affected by nicotine treatment (Fig. 6B). Consistently with the reduced brain dopamine levels in Sph-1 expresing animals, newly eclosed flies that express Sph-1 exhibit decreased levels of tyrosine hydroxylase isoform 1 which is expressed in the dopaminergic neurons throughout the fly´s life. In young flies the higher MW cuticle specific isoform 2, can also be detected; this isoform stops being expressed as soon as the cuticle is melanized and can no longer be detected in aged flies^33^ (Fig. 6C and Supplementary Fig. 4). The aged PD fly model also exhibited reduced tyrosine hydroxylase protein expression compared with control flies (Fig. 6D and Supplementary Fig. 4). Nicotine treatment did not affect tyrosine hydroxylase levels in aged control flies (Fig. 6D and Supplementary Fig. 4). However, nicotine treatment partially recovered the deficiency in dopamine and tyrosine hydroxylase protein levels in Sph-1 expressing flies. In fact, the significant difference observed between the two strains in control conditions (no nicotine treatment) is no longer observed between control and experimental flies due to an increased tyrosine hydroxylase expression in experimental flies after chronic nicotine treatment. This finding would be consistent with the idea that the recovery of brain dopamine levels observed after chronic nicotine treatment (Fig. 6B), could be at least partially explained by the increase in tyrosine hydroxylase protein levels (Fig. 6D).

### Nicotine promotes an increase in survival even when treatment begins in aged animals

**Figure 7:**
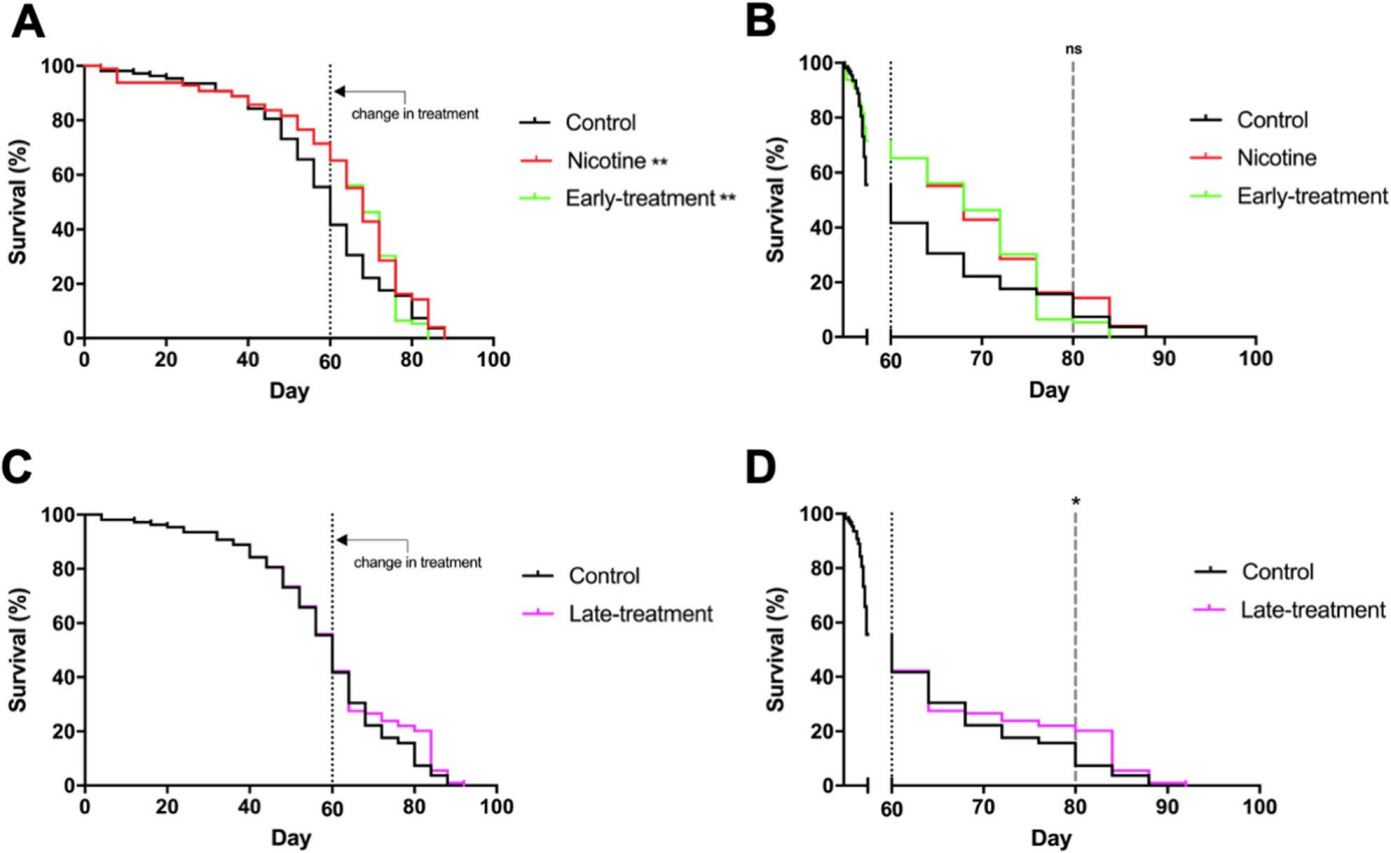
Late in life nicotine treatment increases survival in old flies that express Sph-1. Fifty percent of the population of flies expressing Sph-1 in dopaminergic neurons die by the time they are 60 days old. This time point was defined as the moment to shift treatment. (**A**) Flies that were treated for 60 days with nicotine (and were then transferred to normal food for the rest of their lives), had a significantly longer lifespan compared with untreated animals; a slight reduction in the lifespan of these flies can be observed when compared with animals that were chronically treated with nicotine for their whole life (80 days), however, this reduction was not statistically significant between these two groups. (**B**) Zoom in of the data shown in panel (**A**) after the 60 day time point to better observe the late lifetime effects. (**C**) Animals not treated with nicotine until they are 60 days old and onwards exhibit a significant increase in their lifespan compared with untreated ones. (**D**) Zoom in of the data shown in panel (**C**) the 60 day time point. Statistical analyses were performed using Log-rank (Mantel-Cox) test. **P < 0.01, n = 100 animals per experimental condition. Fisher´s exact tests were used to evaluate the differences at day 80 (*p < 0.05, ns = no significative).

We then decided to determine whether nicotine has a long-lasting effect even if treatment is suspended when flies age. We also studied whether starting the nicotine treatment in older animals is still effective at promoting fly survival. We determined that the half-life of animals that express Sph-1 is 60 days (Fig. 7A); at this age, parkinsonian phenotypes are evident and there is an important decrease in the number of dopaminergic neurons (Fig. 5B). To determine if nicotine has a long-lasting effect even if treatment is suspended, we chronically treated Sph-1 expressing flies for 60 days and then suspended the treatment; in this condition, a significant lifespan extension was observed compared with animals that were never nicotine fed (Fig. 7A,B). Interestingly, files that were not treated with nicotine for the first 60 days of their life and then received nicotine also had a significant extension of their lifespan (Fig. 7C,D). These results indicate that in the PD genotype nicotine has a beneficial effect on survival even when the drug is provided at older age, when the parkinsonian symptoms are already evident. Importantly, this effect appears to be very specific as suspension of the nicotine treatment slightly decreases flies’ survival.

## Discussion

PD is a neurodegenerative disease that has a multifactorial etiology. Previous research has found evidence of genetic and environmental factors that trigger or modulate disease progression. Importantly, only 10% of the cases studied have well-defined genetic causes while the rest are considered sporadic^1^. It has been recognized that α-Syn and Sph-1 have a central role in PD progression^7,34,35^. These proteins appear to be associated to different cellular functions, in particular to the assembly or recycling of presynaptic vesicles. Research on the processes that transform Sph-1 into a toxic protein is likely to be central for our understanding of PD and how neural metabolism contributes to this disease^36–38^.

A limited number of reports exist on the role of Sph-1 in PD progression. There is evidence that the short isoforms of Sph-1 accumulate in post-mortem samples of patients with the disease. In vitro studies show that Sph-1 short isoforms promote protein aggregation and toxicity^12^. Other studies support that the overexpression of Sph-1 or the presence of the mutation R621C in the gene coding for this protein, cause motor impairment and cell damage in PD patients and in a transgenic mice model^39^. A previous work from our laboratory showed that panneurally-expressing Sph-1 flies exhibit motor alterations^21^, which is consistent with the present work where we express this protein only in dopaminergic neurons. Our data is consistent with the hypothesis that flies that exhibit reduced dopamine brain levels are also impaired in their climbing ability. Our results showing alterations in basal and startle-induced locomotion are also in agreement with previous reports demonstrating that flies that express genes associated with PD exhibit several motor and behavioral phenotypes such as centrophobism, bradykinesia and reduction in total distance traveled^21,40,41^. Although most research about PD is focused on neurodegeneration and motor symptoms, there are many other non-motor symptoms that appear early in the disease and are associated to sensory processes such as olfaction, vision, or involve other manifestations like gastrointestinal problems and depression. These non-motor symptoms have a negative impact in the quality of life of patients^42–44^, and are relevant because they are considered early markers of PD. Remarkably, our results show that in young Sph-1 expressing flies it is possible to observe a very characteristic non-motor symptom, olfactory dysfunction, before the onset of motor disabilities. Thus, the Sph-1 PD model share this characteristic phenotype with other animal models of the disease (e.g. the Pink1 mutant flies)^25^, and provides an excellent opportunity to evaluate the effect of potential therapeutic molecules on early stages of PD progression.

In this regard, nicotine is a remarkably interesting molecule, since it is easily available and given its excitatory properties is able to induce neuronal activation; despite the fact it has several negative secondary effects. Nicotine is a chemical already being consumed by a big proportion of the world population and seems to exhibit PD preventive properties. Actually, it has been proposed that people with parkinsonian risk probably discover empirically that nicotine suppresses early-onset tremors and begin to self-medicate with tobacco, thus preventing or delaying the onset of this disease^17,19^. Research from other groups has associated nicotine with protective and/or preventive effects against parkinsonian symptoms; similar protective effects have been observed in mammalian models where dyskinesias are suppressed by nicotine. The understanding of the molecular mechanisms that underlie the protective or preventive effects of nicotine, is an important research avenue where the use of animal or in vitro models could be very useful^18,26,45–49^. Here we decided to advance on this issue, by studying the potential anti-parkinsonian effect of nicotine in flies that express Sph-1^21^. Chronic nicotine treatment was effective at reducing motor parkinsonian phenotypes caused by Sph-1 expression and it did slow down dopaminergic neuron loss. The increase in dopamine levels in the brains of the Sph-1 flies 20 days after nicotine exposure suggests that the protective effect of this drug is via the stimulation of dopamine synthesis and/or release. The neuronal activating properties of nicotine may promote a healthier neuronal metabolism that directly augments dopamine homeostasis. Previous reports have shown that nicotine directly stimulates dopamine release through the activation of nAChRs present in dopaminergic neurons, lowering cytoplasmic dopamine concentrations and probably reducing levels of chelatable iron. Nicotine protective effect is mediated through nAChRs activation as it is blocked by mecamylamine, at least in other models^49^. Lower intracellular dopamine and iron levels induced by nicotine stimulation reduce oxidative stress and therefore decrease the production of dopamine degradation products such as quinones that are known to be toxic. Thus, the intracellular environment becomes less aggressive and proteins that are enriched in Lewy bodies, such as Sph-1 and α-Syn become less prone to aggregate. The unaggregated proteins are therefore unable to sequester tyrosine hydroxylase, and keep playing their normal functions, which include their interaction with the SNARE complex, thus maintaining the ability of the dopaminergic neurons to release dopamine even in a genetic environment that predisposes to PD^50–52^. Increased brain dopamine levels may be explained by de novo synthesis of the bioamine as the enzyme tyrosine hydroxylase exhibits feedback inhibition by the catecholamine. Reducing dopamine intracellular levels might result in less enzyme inhibition and therefore, increased activity of the biosynthetic enzyme. The newly synthesized dopamine is then rapidly transported into presynaptic vesicles by VMAT2, secreted after nicotine stimulation and rapidly recycled by DAT and VMAT into presynaptic vesicles, leading to a net increase in dopamine content^53^.

An interesting finding of our work is that nicotine does not have beneficial effects in control flies; actually, reducing their lifespan and inducing motor impairment in these animals. Our results are consistent with what has previously been reported in Parkin mutant flies where it has been shown that nicotine also promotes survival only in the predisposed genotype. Importantly, in both cases the nicotine effects are dose-dependent^26^. How nicotine differentially affects the control and PD genotypes is outside the scope of this work. However, a possible explanation for the differential effect of nicotine in control and the PD fly model, is that the chemical directly improves cell metabolism and overall functionality in cells. Thus, in metabolically challenged cells such as the dopaminergic neurons that express Sph-1 which contain reduced amine levels, the activation of nicotinic receptors, the consequent depolarization and increase in intracellular calcium enhances dopamine synthesis and release, thus improving dopaminergic function, as discussed above^54,55^. On the other hand, in control non-parkinsonian flies that exhibit normal dopamine levels, nicotine might act as an excitotoxic insult, due to the fact that promoting dopamine metabolism could result in the generation of quinones and an imbalance in the intracellular redox. Further work is needed to advance our understanding on how nicotine differentially affects these genotypes.

Early PD diagnosis is associated to a better prognosis and therapeutic management, which is relevant for a better quality of life of patients. Early PD diagnosis continues to be difficult and once diagnosis is reached, there are very few treatments options for PD patients. L-DOPA continues to be the most widely used treatment for PD and its administration usually begins once the motor symptoms are evident. It is known that this chemical induces tolerance and overtime show reduced effectiveness, making dyskinesias reappear^56,57^. The search of new treatments with fewer side effects continues. Our results show that a protocol for nicotine chronic treatment is beneficial for the PD fly model. Feeding PD flies nicotine and then stopping its consumption still has beneficial effects in fly survival. Notably, a nicotine treatment that begins later in life increases fly survival in the Sph-1 expressing flies. Our results highlight the necessity to keep studying the effects of nicotine in PD models such as the Sph-1 expressing flies and advance on the idea that nicotine could be an important seed molecule for the development of new treatments even when the disease is well advanced.

In conclusion our data show that the PD model by expression of Sph-1 in dopaminergic neurons provides a good opportunity to study the early prodromal stages of PD, while also the late onset symptoms such as neurodegeneration and motor impairment in aged animals. On the other hand, working on this animal model has allowed us to advance on the therapeutic effects of nicotine treatment over several PD-linked features. The protective effect of nicotine appears to be specific for the genotype predisposed to develop a parkinsonian phenotype and provide a hint on the idea that nicotine treatment even in later stages of the disease could be beneficial to patients. Our findings provide new ideas that contribute to a better understanding on the mechanisms underlying the positive effects of nicotine in PD.

## Supplementary information

Supplementary Information.

Supplementary Figure 1.

Supplementary Figure 2.

Supplementary Figure 3.

Supplementary Figure 4.

Supplementary Legends.
