# Supplementary figures and images for: Nicotine suppresses Parkinson’s disease like phenotypes induced by Synphilin-1 overexpression in Drosophila melanogaster by increasing tyrosine hydroxylase and dopamine levels
Source: Sci Rep. 2021 May 5;11:9579. doi: 10.1038/s41598-021-88910-4 (PMC8099903; doi:10.1038/s41598-021-88910-4)

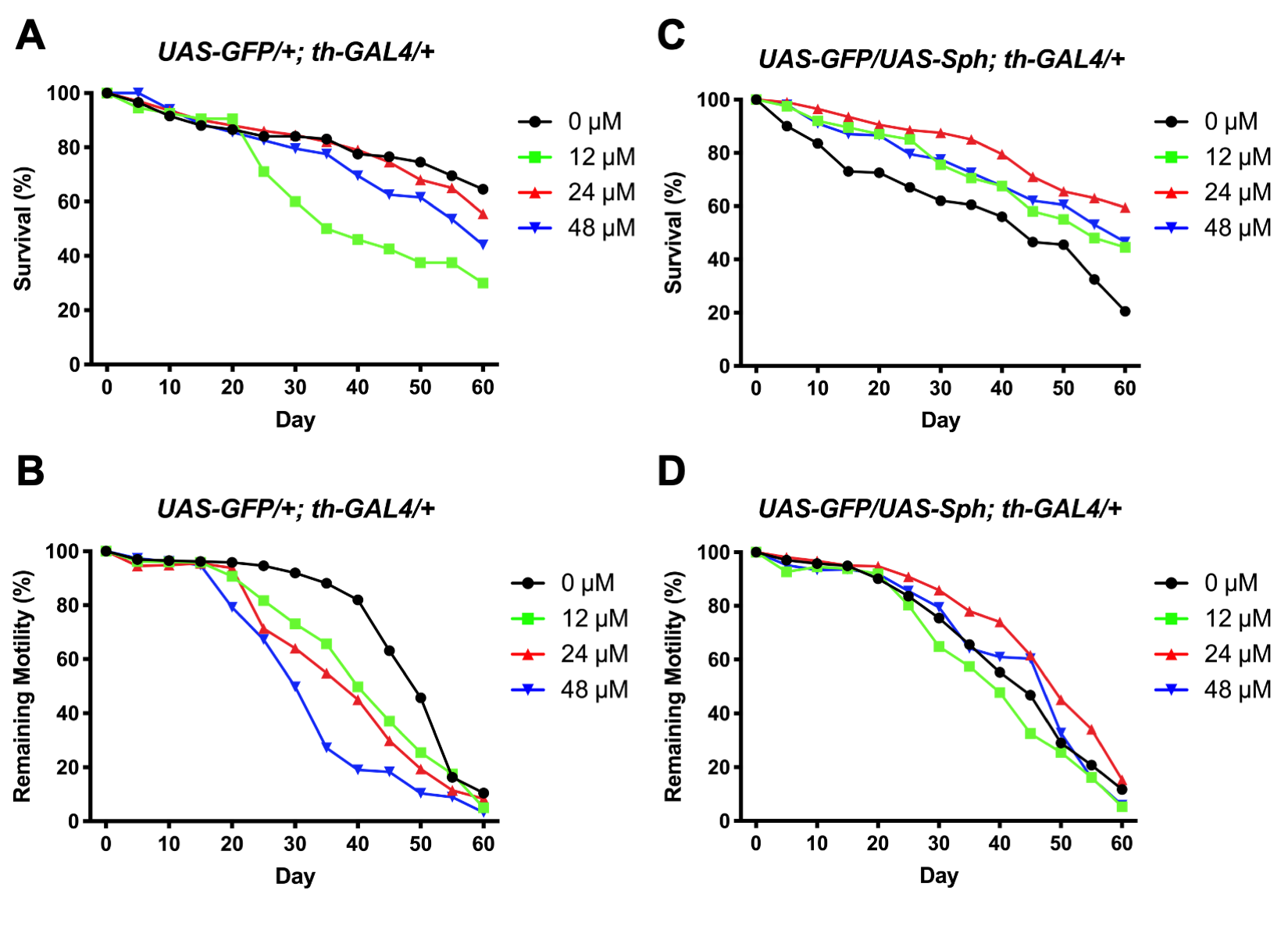

Supplement: Supplementary file 2 — Supplementary Figure 1. [file 41598_2021_88910_MOESM2_ESM.tiff]

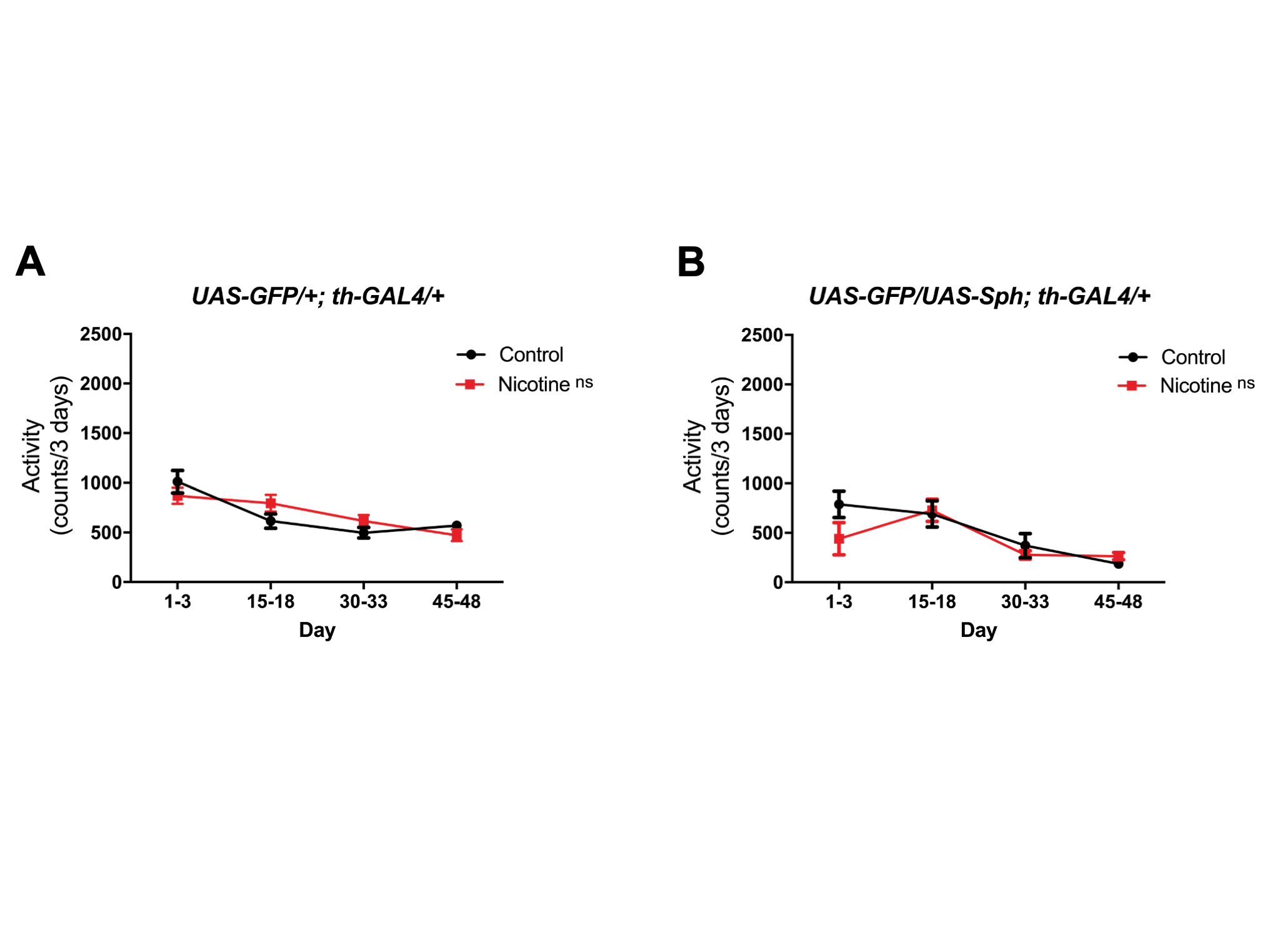

Supplement: Supplementary file 3 — Supplementary Figure 2. [file 41598_2021_88910_MOESM3_ESM.tiff]

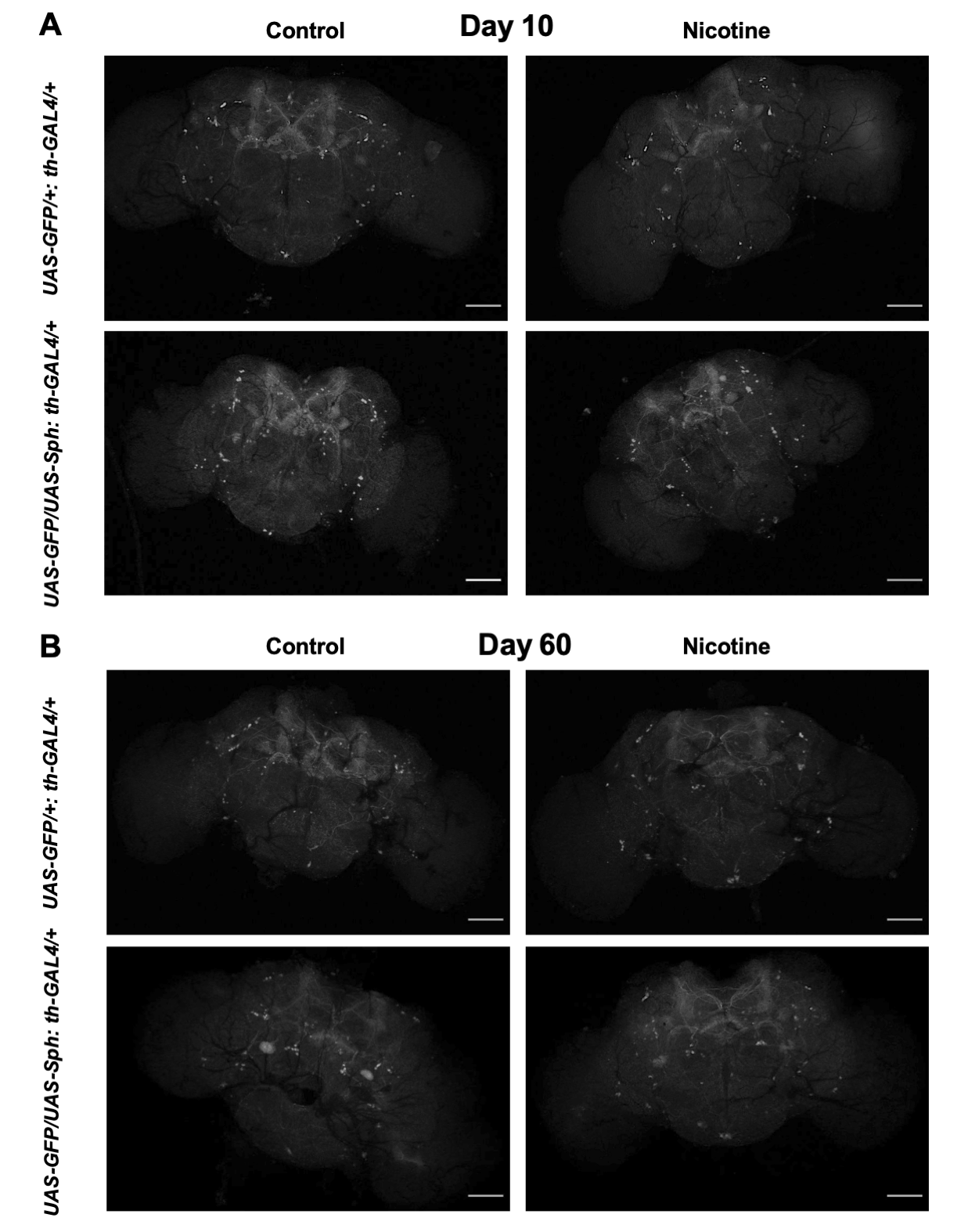

Supplement: Supplementary file 4 — Supplementary Figure 3. [file 41598_2021_88910_MOESM4_ESM.tiff]

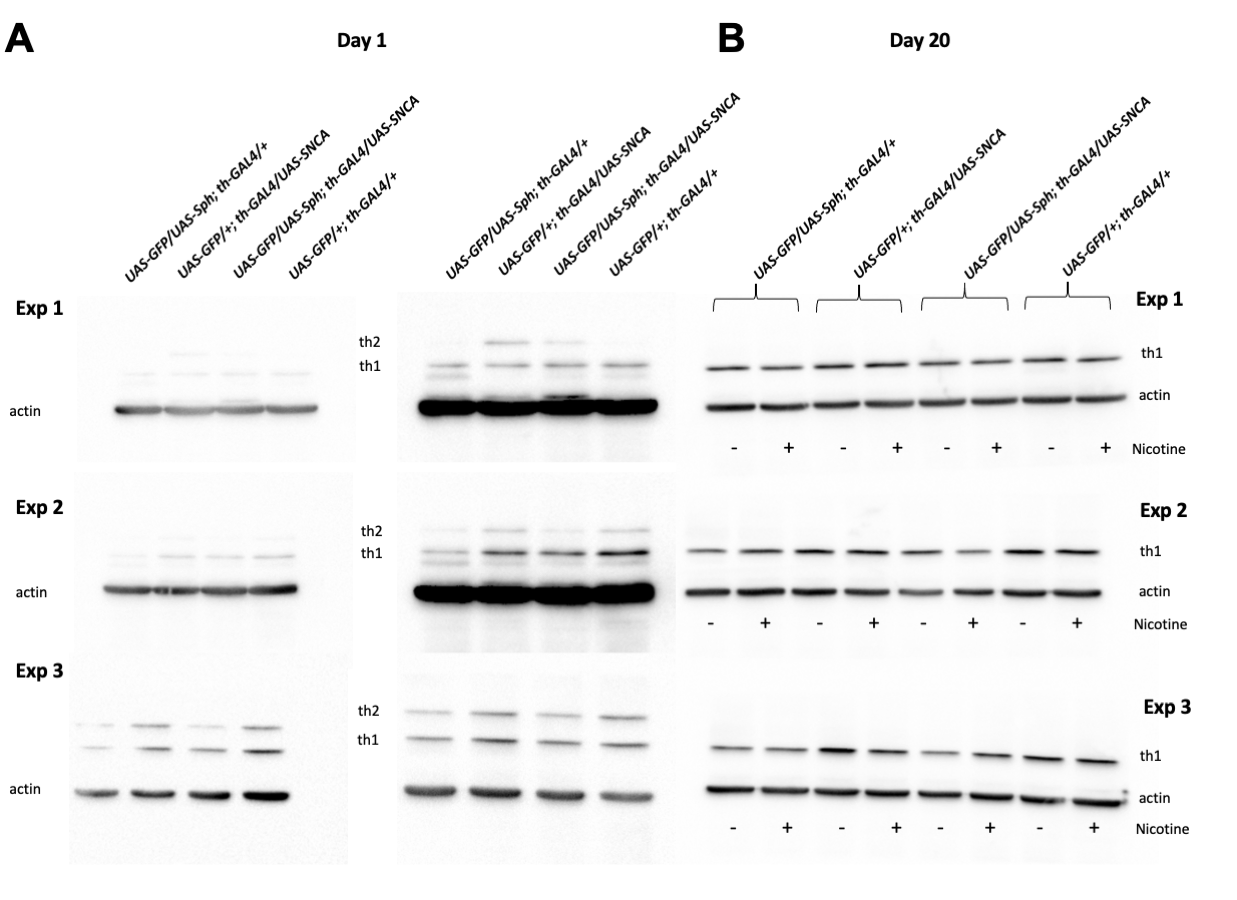

Supplement: Supplementary file 5 — Supplementary Figure 4. [file 41598_2021_88910_MOESM5_ESM.tiff]
