# Supplementary material for: Nicotine suppresses Parkinson’s disease like phenotypes induced by Synphilin-1 overexpression in Drosophila melanogaster by increasing tyrosine hydroxylase and dopamine levels
Source: Sci Rep. 2021 May 5;11:9579. doi: 10.1038/s41598-021-88910-4 (PMC8099903; doi:10.1038/s41598-021-88910-4)
Supplement: Supplementary file 6 — Supplementary Legends. [file 41598_2021_88910_MOESM6_ESM.docx]

**Supplementary Figure 1.­**- **Effect of different nicotine concentrations on lifespan and motility.** Adult males of the control line **(A** and **B)** or males that express Sph-1 in their dopaminergic neurons **(C** and **D)** were reared in nicotine supplemented fly food. Three different concentrations were used. Control animals (*UAS-GFP/+; th-GAL4/+*) showed a reduction in lifespan when treated with nicotine. The flies that express Sph-1 in dopaminergic neurons had longer life expectancy and increased motility when chronically treated with nicotine. The concentration that was most effective was 24 µM and was used in all subsequent experiments.

**Supplementary Figure 2.**- **Activity on dark periods.** **A)** Control animals are not affected by nicotine treatment in spontaneous activity during the dark periods. **B)** Sph-1 expressing animals do not have any change in spontaneous activity during the dark periods. Nicotine treatment neither affects this behavior. Data from flies in control conditions are represented in black, while data from nicotine-treated animals is shown in red. Spontaneous motility significance was evaluated using two-way ANOVA, Tuckey’s post hoc test ns: no significant n=14 animals per group. In all cases experimental animals were treated with 24 µM nicotine.

**Supplementary Figure 3.- Representative maximum projections of confocal images of the *Drosophila* adult CNS of the different genotypes treatments and ages used to quantify surviving neurons as flies age.** A th-GAL4 driver was used to drive the expression of GFP and Sph-1 to dopaminergic neurons. Most of the neurons that express GAL4 in this line are th positive. **A)** Ten-days old representative brains of the nicotine treated and untreated control and experimental lines (*UAS-GFP/+;th-GAL4/+* and *UAS-GFP/UAS-Sph;th-GAL4/+* respectively) B) Sixty-days old representative brains of the nicotine treated and untreated control and experimental lines (*UAS-GFP/+;th-GAL4/+* and *UAS-GFP/UAS-Sph;th-GAL4/+* respectively). An evident reduction of dopaminergic cells can be observed in 60 days old untreated experimental brains. Confocal acquisition parameters were established in such a way that all the mayor and most characteristic dopaminergic clusters were detectable in the newly eclosed control flies. Once these acquisition conditions were defined they were used for dopaminergic neuron quantification in all the other experimental conditions. There is a slight probability that some of the few none dopaminergic cells trapped in this line were included in the quantification but as mentioned, they are a very small minority and being non dopaminergic they should be a constant. Scale bar 100 µM.

**Supplementary Figure 4.**- **Western blots used for quantification of tyrosine hydroxylase.** All densitometric experiments were performed in the same blot. In interest of clarity we only show in figure 5 the relevant lanes (th and actin for treated and not treated flies that express or not Sph1). In this figure we provide whole unedited membranes used for the densitometric experiments reported in this work. Membranes show th1/th2 and actin expression of nicotine treated and untreated animals that express α-Syn and Sph-1 **A)** One day old fly heads western blots. Neural (th1) and cuticle (th2) tyrosine hydroxylase isoforms can be detected only th1 was used for quantification. **B)** Twenty days old fly heads Western blots. Neural (th1) is expressed throughout life while the th2 tyrosine hydroxylase isoform is only expressed during cuticle maturation and later cannot longer be detected. Actin was used as loading control.
